# Insulin‐like growth factor binding protein‐4: The quest for the breakthrough biomarker in pulmonary arterial hypertension

**DOI:** 10.1002/pul2.12252

**Published:** 2023-06-20

**Authors:** Rodolfo A. Estrada, Sandeep Sahay

**Affiliations:** ^1^ Division of Pulmonary Diseases and Critical Care Medicine UT Health San Antonio Texas USA; ^2^ Division of Pulmonary, Critical Care & Sleep Medicine Houston Methodist Hospital Houston Texas USA

**Keywords:** biomarker, pulmonary hypertension, severity, survival

Despite the advances made in diagnosis, treatment, and management of pulmonary arterial hypertension (PAH), survival has remained poor. Contemporary registries have shown survival estimates of 79% and 71% at 3 and 5 years, respectively.^1^, ^2^ PAH subtypes have varying symptom progression, hemodynamics, and survival profiles.^3^ There has been a recent surge of interest in exploring less invasive approaches to identify factors that contribute to early diagnosis and prognosis. These elements can also aid in guiding therapy and serving as surrogate endpoints in future trials. An extensive array of biomarker attributes has been studied including cell structure, circulating cell population, genetic markers and epigenetics, proteomics, and microRNAs. N‐terminal pro‐brain natriuretic peptide (NTProBNP) is the only biomarker extensively studied and currently being used to guide management and prognosis.^4^ However, it is not a specific marker for pulmonary vascular disease and does not directly reflect injury to the pulmonary vasculature but rather right ventricular dysfunction; additionally, it is commonly confounded by other factors such as renal dysfunction and left heart disease.^5^

The characteristics that contribute to vascular remodeling have been the primary target of new discoveries, including angiogenic factors, vasoactive ligands, and inflammatory cytokines.^6^ Energy metabolism pathways and their association with cell proliferation and growth have been related to PAH, in particular the insulin‐like growth factor (IGF) axis pathways.^6^ High‐throughput plasma proteome analysis in PAH patients has identified proteins that correlate to survival, identifying the insulin‐growth factor binding protein‐1 (IGFBP‐1) as one of them.^6^ There are six of these binding proteins and circulating IGFBP‐2 levels have been found to not only have correlation with pulmonary fibrosis progression and response to treatment, but more recently, also to PAH severity and mortality with value as prognostic marker.^7^ Prior studies on IGFBP‐4 have been focused on its effect on cellular mitosis and proliferation, as well as its role as antiangiogenic, antitumorigenic, and cardiogenic growth factor.^8^ IGFBP‐4 has also been related to defects in cell differentiation and growth, hypoxia signaling, and fibrosing mechanisms^9^ (Figure 1).

**Figure 1:**
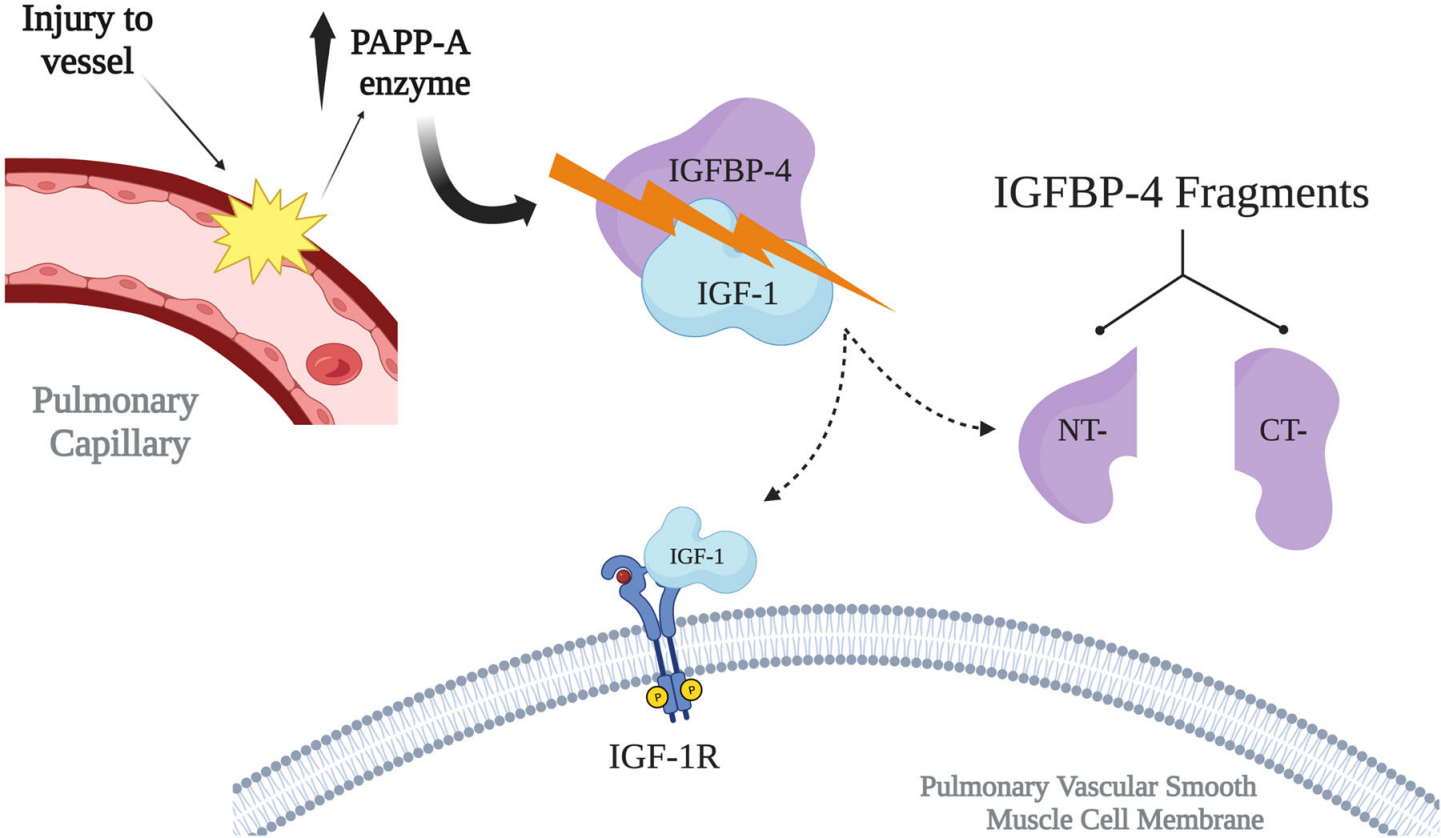
Schematic illustration of proposed mechanism of elevated levels of IGFBP‐4 related to pulmonary arterial hypertension (PAH). Injury to the pulmonary arteries leads to increased expression levels of the PAPP‐A enzyme by the pulmonary artery smooth muscle cells (PASMC). This protease is responsible of IGF‐dependent cleavage of IGFBP‐4, resulting in 2 fragments: N‐(NT) and C‐terminal (CT). Upon activation, insulin‐like growth factor (IGF) is released near the IGF‐1 receptor which then contributes to pulmonary artery smooth muscle cells (PASMC) migration, growth, and proliferation.

In this issue of pulmonary circulation, Torres et al.^10^ report the findings of a multicenter study of patients with PAH [John Hopkins—JHPH (*n* = 127), National Biological Sample and data repository for PAH—PAHB (*n* = 2579), and Vanderbilt—VLPH (*n* = 127)]. In these patients, IGFBP‐4 levels were correlated with PAH severity and survival during a median follow‐up of 4.2–5 years. The results were contrasted with a control group (*n* = 90), which was noted to be smaller in proportion to the studied groups. However, the population was overall well characterized. This study explored IGFPB‐4 levels in serum using ELISA methods, and in cultured pulmonary artery smooth muscle cells (PASMC) and pulmonary artery endothelial cells (PAEC) to know whether these were a source of IGFBP‐4 in lung tissues by measuring the mRNA expression levels.

The median age was 62, 52, and 55 years old with a predominantly female population at 85%, 79%, and 81% in the JHPH, PAHB, and VLPH cohorts, respectively. The predominant PAH subtypes in the cohorts were idiopathic pulmonary arterial hypertension (IPAH) [between 36%–49%] and associated pulmonary arterial hypertension (APAH) [48%–64%]. Baseline 6‐min walk distance (6MWD) and severity assessed by hemodynamics were noted to be similar across cohorts, except for higher mean pulmonary arterial pressure (mPAP) and pulmonary vascular resistance (PVR) in the PAHB cohort.

The main finding was that levels of IGFPB‐4 were significantly elevated in PAH patients compared to controls (*p* < 0.0001). To differentiate PAH patients from controls, a serum IGFBP‐4 value of 352.2 ng/mL was established in the test cohort (JHPH). Circulating IGFBP‐4 levels were also significantly increased in the validation cohort (PAHB) and at baseline in the longitudinal cohort (VLPH). Levels of IGFPB‐4 were dichotomized by the median in all cohorts, and higher levels were noted to be associated with worse PAH survival, progression, and severity, defined by a decreased 6MWD, New York heart association functional class, Reveal Lite 2.0 score, and higher right atrial pressures.

Subgroup analysis revealed that regardless of PAH subtype, above median IGFBP‐4 levels were associated with worsening survival rates. Connective tissue‐associated PAH (CTD‐PAH) had the highest serum level of IGFPB‐4, and congenital heart disease (CHD‐PAH) had the lowest. CHD‐PAH was also noted to have higher PVR and mPAP levels of all subgroups evaluated. Idiopathic PAH (IPAH) had the worst survival and highest risk classification profile despite not having the highest IGFBP‐4 levels. This study also showed an increased concentration and mRNA expression of IGFBP‐4 in cultured PASMC and PAEC of PAH patients, which highlights that the lungs are a potential source of IGFBP‐4 secretion. However, its relationship to PAH pathophysiology is still unknown.

The authors suggest the use of IGFBP‐4 as a potential biomarker to assess PAH disease severity, progression, and prognosis. But is IGFBP4 ready for primetime? The results are attractive, but we caution certain limitations. For the CTD‐PAH cohort, an ideal control group would have been patients with CTD but without PAH. Another point to highlight is that these cohorts have a significant number of outliers, particularly for the PAHB cohort. There are some patients that fall at or below the 25th interquartile range (IQR) and are at the same level of the median for the control group. IPAH had the worst survival and risk profile, despite CTD‐PAH having the highest IGFBP‐4 levels; and CHD‐PAH had the highest mPAP and PVR with the lowest IGFPB‐4 levels. These findings suggest that the specific IGFBP‐4 cutoff values may vary depending on disease severity and associated PAH subgroup. The severity, and associated mortality risk, was assessed with the REVEAL Lite 2.0 risk score; however, only half the subjects had complete risk data, and most were classified as low risk. Nevertheless, when risk was assessed by PAH subtype, higher IGFBP‐4 values were associated with a higher REVEAL Lite 2.0 risk. The PAHB cohort had the lowest mortality compared to the other cohorts, and it was unclear if this was driven by PAH or all‐cause mortality.

Studies have shown different biomarkers associated with clinical outcomes and mortality in PAH, but generalization across all PAH patients has been challenging given the disease heterogeneity.^3^ Circulating IGFBPs are associated with other cardiopulmonary diseases and shown to have prognostic and therapeutic implications, as shown in a study evaluating IGFBP‐2 levels to be reduced by antifibrotic and/or anti‐inflammatory medications.^7^ Torres et al. showed an encouraging strong survival correlation when analyzing NT‐proBNP and IGFPB‐4 levels stratified by median levels, given that NT‐proBNP is currently the most validated biomarker for PAH.

Strong interest has been developed through the advent of molecular science and artificial technology to further clarify potential pathways to improve diagnosis, prognosis, and available therapies for PAH patients. Novel biomarkers have proven useful in prognosticating outcomes independent of the REVEAL score with potential to predict response to therapy, particularly in CTD‐PAH.^3^, ^6^ Early high‐throughput analysis of plasma proteome identified a nine‐protein panel in PAH patients that predicts survival independent of NT‐proBNP, with IGFBP1 included as one of the proteins in the panel. A higher risk score calculated by the protein panel, was associated with higher individual risk, which improved the prediction of existing clinical risk models, like REVEAL. Furthermore, failure to respond to therapy once it was started, was identified as changes in the panel score of these patients. The prognostic capacity of this protein panel was able to identify patient subgroups at higher risk for events (death or transplant).^6^

IGFBP‐4 appears to be an exciting new biomarker that is associated with PAH disease severity and survival, as shown in multiple heterogenous PAH cohorts. Cautious interpretation is still advised given the data was collected retrospectively and that the mechanisms of action and related specific PAH pathobiology is still unclear. This study provided guidance that IGFBP‐4 assessment in PAH patients adds to the armamentaria of potential novel biomarker pathways that can correlate with severity and survival. Further research is encouraged to determine how these findings can be incorporated in panels or algorithms for early diagnosis and prognosis, to help guide therapy and potentially serve as surrogate endpoint in future trials of this devastating and fatal disease. We congratulate the investigators for the discovery of novel biomarkers, such as IGFPB‐4, which may lay a bright future ahead for the PAH research, clinical, and patient community.

## AUTHOR CONTRIBUTIONS

**Rodolfo A. Estrada**: Prepared initial draft and participated in revisions. **Sandeep Sahay**: Concept, critical revisions, editing, and owner of this manuscript.

## CONFLICTS OF INTEREST STATEMENT

Rodolfo A. Estrada: Advisor for Janssen. Sandeep Sahay: Grants/Research Support: Research grant from United Therapeutics; Consultant: United Therapeutics, J&J, Bayer, Gossamer Bio, Liquidia technologies, Keros; Speaker's Bureau: Janssen, United Therapeutics (honorarium received by Houston Methodist Hospital Foundation); Clinical trial support from United Therapeutics, Janssen, Gossamer Bio, Altavant Sciences, Liquidia technologies, Novartis, Keros.

## ETHICS STATEMENT

Not applicable.
